# “Side effects--part of the package”: a mixed methods approach to study adverse events among patients being programmatically treated for DR-TB in Gujarat, India

**DOI:** 10.1186/s12879-020-05660-w

**Published:** 2020-12-02

**Authors:** Arjunkumar Jakasania, Kalpita Shringarpure, Dixit Kapadia, Radhika Sharma, Kedar Mehta, Arpit Prajapati, Soundappan Kathirvel

**Affiliations:** 1 Department of Community Medicine, MGIMS, Sewagram, Wardha, Maharashtra India; 2 Department of Preventive and Social Medicine, Medical College Baroda, Vadodara, Gujarat India; 3 District Tuberculosis officer, Ahmedabad, Gujarat India; 4 Department of Community Medicine, BJ Medical College, Ahmedabad, Gujarat India; 5 GMERS medical college, Gotri, Vadodara, Gujarat India; 6 Department of Community Medicine, GCS Medical College, Ahmedabad, Gujarat, India; 7 Department of Community Medicine and School of Public Health, Post Graduate Institute of Medical Education and Research (PGIMER), Chandigarh, India

**Keywords:** Adverse drug event, Drug resistant tuberculosis, Operational research, Incidence, India

## Abstract

**Background:**

High rates of Adverse Events (AEs) during treatment is one of the leading causes of unsuccessful treatment outcomes among patients with drug resistant tuberculosis (DR-TB). However, information related to AEs is not systematically collected and managed under programmatic setting. The present study assessed the a) incidence and pattern of adverse events in first three months of DR-TB treatment initiation; b) treatment seeking behaviour for AE management; and c) explore the challenges in seeking treatment and reporting AEs.

**Methods:**

This mixed methods study included all patients diagnosed and initiated on treatment under RNTCP during July–September 2018 at Ahmedabad DR-TB centre. The patients were interviewed telephonically and assessed for all AEs experienced by them. In-depth interviews and key-informant interviews were conducted among patients, DOTS supervisors and programme staff (treatment supervisors, medical officer and district program managers).

**Results:**

Total 207 AEs were reported by the 74 DR-TB patients. All patients experienced at least one AE during initial treatment period. Incidence rate of AEs (experienced) was 3.11 (1st month-4.6, 2nd month-2.7, 3rd month-2.02) per 100 person days. Of the 207 AEs, gastro-intestinal (59, 28.3%), ophthalmic (32, 15.4%) and otolaryngology (25, 11.9%) system related AEs were commonly experienced. Treatment was not sought in two-fifths of the AEs. Themes and sub-themes related to challenges in treatment seeking or reporting of AEs were 1) Patient related-Misconceptions, accessibility and affordability of management, lack of counselling support, stigma and discrimination, and past treatment experience; 2) Health system related- lack of guidelines and training for AE management, 3) Poor coordination between hospital and tuberculosis centre.

**Conclusion:**

The incidence of AEs was high among patients with DR-TB in the first three months of treatment and treatment seeking/reporting was low. Adequate health education and counselling of the patient and orientation of the health systems is the need of the hour. An efficient real-time reporting and management of AE should be developed and tested for effective DR-TB control.

**Supplementary Information:**

The online version contains supplementary material available at 10.1186/s12879-020-05660-w.

## Background

Tuberculosis (TB) is the leading infectious cause of death and still continues to be a major public health problem. The emerging issue of Drug-resistant Tuberculosis (DR-TB) due to inappropriate treatment regimens, poor quality drugs and inadequate or incomplete intake of first-line Anti-TB drugs has worsened the situation; making its management quite a challenge [1]. Globally, an estimated 558,000 people infected with *Mycobacterium tuberculosis* were resistant to the most effective first-line drug i.e., rifampicin (RR-TB), and of these, 82% had multidrug-resistant TB (MDR-TB) in 2017. Similarly, an estimated 3.5% of new cases and 18% of previously treated cases had MDR/RR-TB globally and India has nearly one fourth of the estimated cases [2].

India, a high burden DR-TB country rolled out the programmatic management of drug resistant TB (PMDT) in 2007 and covered all parts of the country by 2013 [3]. Over a period, the country implemented the World Health Organization’s (WHO) ambulatory care model and changed from criteria-based drug sensitivity testing (DST) to universal DST to detect and treat the DR-TB to address the DR-TB burden. Now, patients with DR-TB are treated with various DR-TB regimens and a shorter DR-TB regimen (based on Bedaquiline and Delaminid) has also been introduced under expanded access in the country. This is due to low successful treatment outcomes (47%) with the conventional regime and vice versa with the shorter regime [4].

Higher proportion of patients with DR-TB are encountering adverse events (AEs) due to long duration of treatment, use of second line anti-tuberculosis drugs, multi-drug use, long duration of injections and other patient related characteristics [5]. It was reported that 20–100% of patients with DR-TB had experienced at least one AE involving gastro-intestinal tract, hepato-biliary, neurological, oto-vestibular, eye, musculoskeletal and other systems; with symptoms ranging from mild to severe and some serious adverse reactions [6–9]. However, anecdotal evidence has suggested that the reporting of AEs by patients is low; this can lead to progression in severity of the AEs and impact treatment outcome. Studies have reported the association of AEs with poor medication adherence and unsuccessful treatment outcomes like loss to follow up, treatment failure and death [10, 11].

There is a large variation observed between studies reporting the number of events and proportions of DR-TB patients with AEs, as most of the studies relied on medical record data abstraction [8–10, 12–15]. These recorded rates could be an underestimation; especially in the context of developing countries like India, where the primary programmatic focus is on diagnosis and initiation of treatment rather than identification, management and reporting of all AEs by the health systems and poor treatment seeking behavior of patients. Low reporting of AEs has its own consequences for the patients; as an unreported AE is often an unmanaged AE.

As there is limited information available on the exact burden of AEs experienced by the patients with DR-TB in Indian context, we assessed the incidence and pattern of AEs experienced by patients with DR-TB, their health seeking behavior and factors associated with non-reporting of AEs under programmatic setting.

## Methods

### Study design

A mixed methods study (sequential explanatory design) with quantitative-retrospective cohort study through record review and patient interviews and qualitative-descriptive design was used. The qualitative findings were used to supplement the quantitative data.

### Study setting

#### General setting

The study was conducted in Ahmedabad district of Gujarat state, India. There are 34 DR district tuberculosis centers (DTC) in the state which have notified 2266 DR-TB cases in the year 2018 [16]. Treatment success rate for patients with DR-TB was reported to be 43% and loss to follow up was 15% in the year 2017. The Ahmedabad district (including Ahmedabad municipal area and rural area) has a population of 7,045,314 including 5,633,927 population of Ahmedabad city, which makes it the most populous city in Gujarat and the fifth most populous in India [16, 17].

##### Ahmedabad DR-TB center

The DR-TB Centre in Ahmedabad is situated in State TB demonstration and training centre (STDC), Civil Hospital campus, Ahmedabad. The state nodal DR-TC and Intermediate state reference laboratory (IRL) is also situated within the campus. It has facility for solid culture and drug susceptibility testing (DST), liquid culture, Line probe Assay (LPA) and Cartridge Based Nucleic Acid Amplification Test (CBNAAT). Patients diagnosed with DR-TB are put on conventional treatment regimen, Universal drug sensitivity guided treatment regimen and Bedaquiline containing treatment regimen. There is a TB and Chest ward which is attached with the centre with advanced treatment facilities [16].

### Study site

DR TB Centre attached to tertiary care hospital in Central Gujarat.

### Study population

For quantitative study, all patients initiated on DR-TB treatment between 1st July to 30th September 2018 and having completed the first three months of treatment under the Revised National Tuberculosis Control Program (RNTCP) at the DR-TB Center, Ahmedabad, Gujarat were included in the study. Interviews were conducted in the fourth month of their treatment. Those patients who were “lost to follow-up” were also included irrespective of ‘treatment taken’ duration. Those patients a) who did not consent for the interviews; b) were transferred out to other district DR-TB center and c) on Bedaquiline regimen were excluded. (The study was conducted in a DR-TB centre which was also the country’s first demonstration/pilot study site for Bedaquiline drugs. During this regimen, there was a system of follow up of patients by dedicated counsellors for patients on Bedaquiline treatment. Hence, the objective of the study to know the proportion of AEs unreported to the program might not be fulfilled in this group).

For the qualitative component, in-depth interview (IDI) was conducted among selected patients and DOTS providers using convenience sampling method. Key-informant interview (KII) was conducted among medical officers, DR-TB supervisor and district programme managers. Twenty patients with DR-TB (14 male and six female) and eight DOTS providers (six Urban, two Rural), two medical officers and two DOTS plus supervisors and senior TB treatment supervisors (one from rural and urban each) and two programme managers at district tuberculosis centre were interviewed.

### Data collection and analysis

A list of patients with DR-TB, who were diagnosed and/or initiated on treatment during 1^st^July 2018 to 30th September 2018 were obtained from DR-TB center. Further, relevant information like name, address, and contact number, date of initiation of treatment, drug resistance status, name of designated DOTS provider and Senior TB supervisor and data on AEs were abstracted from treatment cards. The patients were telephonically interviewed once at end of third month of treatment initiation for AEs experienced by them monthly in the last three months using a semi-structured questionnaire developed by team of investigators for this study. The details collected during the interview were noted down using electronic data collection tool (KoBo Collect v1.14) [18]. If any AE was experienced by them in past three months, details like the month of AE occurrence, classification of system involved (including the symptoms) as per International Council for Harmonisation of Technical Requirements for Pharmaceuticals for Human Use-ICH classification [19] with expert recommendation which has been used in various studies [5, 20]; and whether treatment was sought for the reported AE were noted. Socio-demographic characteristics like age, gender, residence (urban/rural), occupation, education and clinical characteristics like co-morbidities (HIV, Diabetes mellitus and other chronic conditions) addiction (tobacco and alcohol) and previous history of TB were collected. The information related to healthcare provider from whom patient took daily injections (Auxiliary nurse midwife/qualified Private practitioner / unqualified private practitioner/ primary health centre-medical officer/other) was also obtained.

The data extracted through record review were entered in the excel. The data collected through the electronic data capture tool was downloaded, merged with extracted data in excel and exported to EpiData version 2.2.2.182 for analysis (EpiData Association, Odense, Denmark). Incidence of AEs has been reported as AE per 100 person-days, per month; and for total duration of three months of initial treatment. Number and proportion were calculated for gender, occupation, residence, per capita income, characteristics of the patients like HIV status, Diabetes Mellitus status, addiction (tobacco, alcohol), past history of TB, patients with DR-TB on treatment experienced AE, pattern of AEs, AEs reported and its pattern, type of healthcare facility and healthcare providers contacted for management of AEs. Age was reported in mean (Standard deviation). For analytical statistics, Chi square test applied with Yate’s correction where applicable. A *p*-value of ≤0.05 was considered statistically significant. The findings have been reported using strengthening the Reporting of Observational studies in Epidemiology (STROBE) guidelines [21].

#### For qualitative data

The principal investigator, trained and experienced in qualitative research methods conducted IDIs among patients after taking their consent during their quantitative interview, using an interview guide developed by team of investigators for this study. The interview explored the views and experiences of the patients related to DR-TB treatment and AEs. Similarly, this also explored their treatment seeking behavior and perspective on facilitators and barriers of seeking medical assistance from healthcare providers. IDIs and KII were also conducted among purposively selected health care providers using interview guide. Interviews were conducted in local language and each interview lasted for nearly 15–20 min. Verbatim notes were taken during the interview. Transcripts were made on the same day of the IDI based on the verbatim notes by co-field investigator. The transcripts obtained were compiled and the PI read the transcripts to become familiar with the data. Manual descriptive content analysis was used to analyze the transcripts. It was reviewed by a second investigator (KS) trained in qualitative research method for interpretive credibility and reducing bias. The decision on coding rules and theme generation were done by using standard procedures and in consensus [22]. Any difference between the two were resolved by discussion. The findings have been reported by using ‘Consolidated Criteria for Reporting Qualitative Research (COREQ) guidelines [23]. Operational definitions have been described in supplementary Table A.

## Results

### Quantitative

**Fig. 1:**
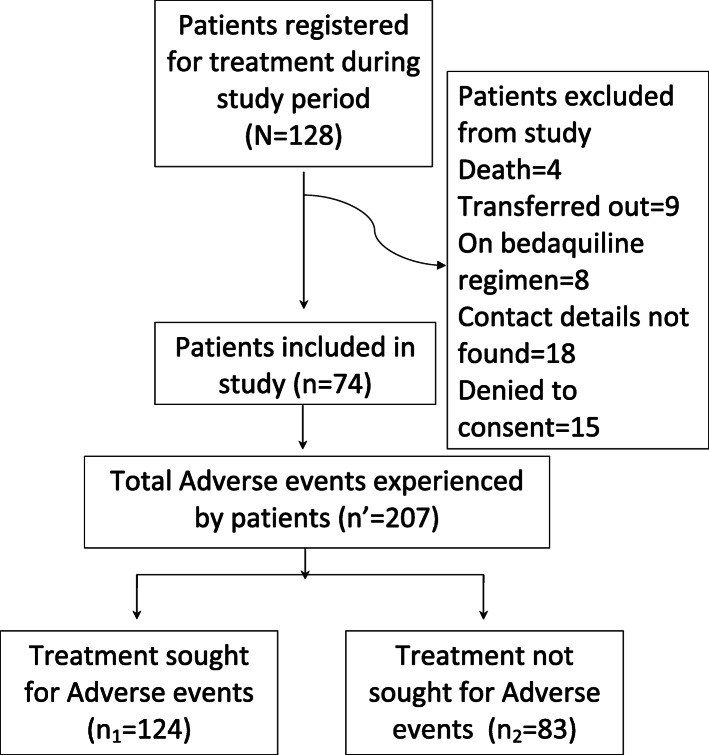
Flow diagram of treatment seeking pattern for AE management of patients initiated on MDR/RR-TB intensive phase treatment (July–September 2018) under the RNTCP at the DR-TB Centre, Ahmedabad, Gujarat

During July–September 2018, a total of 74 eligible patients were interviewed among 128 patients registered and put on treatment at the study site (Fig. 1). The mean (SD) age of participants was 32.4 (+ 13.6) years. Of the 74 patients, 44 (59.5%) were male; 46 (62.2%) were educated up to secondary school; 54 (73%) belonged to below poverty line; 60 (81.1%) were residing in urban area; and 64 (86.5%) had tuberculosis in the past. The socio-demographic, behavioral and clinical characteristics of the participants are given in Table 1. A total of 22 (29.7%) patients were tobacco users (either smokeless or smoking) and 24 (32.4%) reported presence of at least one comorbidity.

**Table 1:** Socio-demographic, behavioral and clinical characteristics of patients with DR-TB initiated on treatment under the RNTCP at Ahmedabad DR-TB Center, Gujarat from July–September 2018

Characteristics	***N*** = 74	(%)
Mean (SD) age in years	32.4 (+ 13.6)	
**Gender**		
Male	44	(59.5)
Female	30	(40.5)
**Education**		
Illiterate	13	(17.6)
Up to secondary school	46	(62.2)
Above secondary school	15	(20.2)
**Occupation**		
Home-maker	05	(06.7)
Unemployed	15	(20.0)
Employed	54	(73.3)
**Area of residence**		
Rural	14	(18.9)
Urban	60	(81.1)
**Socio-Economic Status**		
Above Poverty Line	17	(23.0)
Below Poverty Line	54	(73.0)
Not known	03	(04.0)
**Addiction**		
No addiction	55	(74.3)
Tobacco chewing	13	(17.6)
Smoking	09	(12.2)
Alcohol	05	(06.8)
No answer	03	(04.1)
**Co-morbidities**		
None	50	(67.6)
Renal disease	12	(16.3)
Cardiovascular system	10	(13.5)
Diabetes	8	(10.8)
HIV	6	(8.1)
Liver disease	04	(05.4)
Others ^a^	04	(05.4)
**Previous TB treatment**		
Yes	64	(86.5)
No	10	(13.5)
**Drug resistance**		
Mono resistance Isoniazid	24	(32.4)
Multi Drug resistance	50	(67.6)
**Health care facility opted for injectables**		
Public healthcare	32	(43.2)
Private health care	32	(43.2)
Non-injectable regimen	10	(13.6)

^a^ Hypothyroidism (2) and Paraplegia (2)

**Fig. 2:**
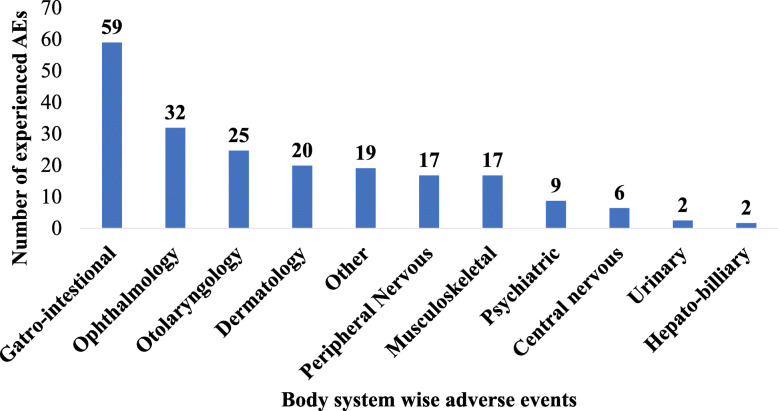
Number of AEs experienced by patients initiated on DR-TB treatment between 1st July to 30th September 2018 under the RNTCP at the DR-TB Center, Ahmedabad, Gujarat. *Other include AE involving multiple organ systems

**Fig. 3:**
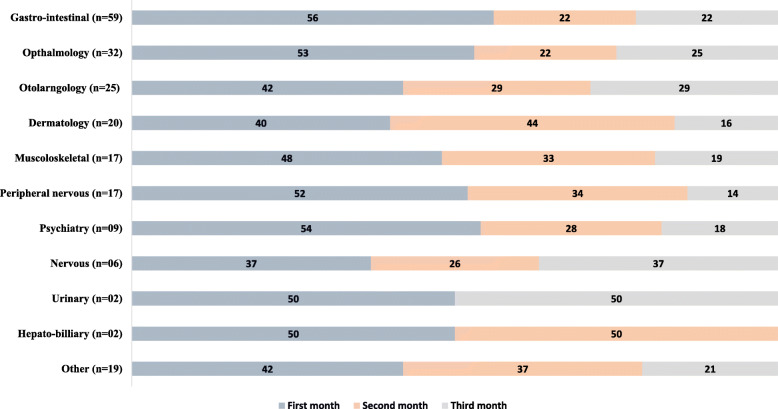
Proportion of AEs as per month of occurrence and organ system involved among patients initiated on DR-TB treatment between 1st July to 30th September 2018 under the RNTCP at the DR-TB Center, Ahmedabad, Gujarat. *Other include AE involving multiple organ systems

All 74 patients experienced at least one adverse event during the first three months of treatment and a total of 207 events were reported. The incidence rate of AEs (per 100 person days) during the study period was 3.11 (4.6, 2.7 and 2.02 for the first, second and third month from initiation of treatment respectively). System wise AEs have been described in Fig. 1. AEs related to gastro-intestinal, ophthalmic and otolaryngology were the the most commonly experienced AEs, occurring among 59 (28.3%), 32 (15.4%) and 25 (11.9%) of the participants, respectively. The most common symptoms under each systems have been described in the supplementary Table B. The pattern and month wise distribution of AEs is depicted in Fig. 2. Proportion of AEs as per month of occurrence and organ system involved among patients is depicted in Fig. 3.

Of the 207 AEs experienced by patients, treatment was sought for 124 (61.7%) AEs (Fig. 1). Patients were categorized as ‘having sought treatment’ if they reported more than 50% of their experienced AEs, ‘not having sought treatment’ if they reported less than 50% of their experienced AEs. None of the patient characteristics were significantly associated with the patient not seeking treatment (Table 2).

**Table 2:** Socio-demographic, behavioral and clinical characteristics associated with treatment seeking behavior for Adverse events by patients initiated on DR-TB treatment under the RNTCP at Ahmedabad DR-TB Center, Gujarat from July–September 2018

Characteristic	Treatment sought		Treatment not sought^b^		Total		***p*** value
	n	(%)	n	(%)	n	(%)	
Total	43		31		74		
**Sex**							
Male	26	(59.1)	18	(40.9)	44	(59.5)	0.83
Female	17	(56.7)	13	(43.3)	30	(40.5)	
**Education**							
Illiterate	12	(92.3)	1	(7.7)	13	(17.6)	0.06^a^
Up to secondary	24	(52.2)	22	(47.8)	46	(62.2)	
> Secondary	7	(46.7)	8	(53.3)	15	(20.2)	
**Occupation**							
Home-maker	3	(60.0)	2	(40.0)	5	(6.7)	0.91^a^
Unemployed	8	(53.3)	7	(46.7)	15	(20.0)	
Employed	32	(59.3)	22	(40.7)	54	(73.3)	
**Residence**							
Rural	9	(64.3)	5	(35.7)	14	(18.9)	0.82
Urban	34	(56.7)	26	(43.3)	60	(81.1)	
**Socio Economic Status**							
Above poverty line	9	(52.9)	8	(47.1)	17	(23.0)	0.94 ^a^
Below poverty line	32	(59.3)	22	(40.7)	54	(73.0)	
Not known	2	(66.7)	1	(33.3)	3	(4.0)	
**Drug resistant**							
Mono Isoniazid	15	(62.5)	9	(37.5)	24	(32.4)	0.76
Multidrug resistant	28	(56.0)	22	(44.0)	50	(67.6)	
**Past history of TB**							
Yes	38	(59.4)	26	(40.6)	64	(86.5)	0.57
No	5	(50.0)	5	(50.0)	10	(13.5)	
**Co-morbidities**							
Present	11	(45.8)	13	(54.2)	24	(32.4)	0.40
Absent	32	(64.0)	18	(36.0)	50	(67.6)	
**Addiction**							
Yes	11	(57.9)	8	(42.1)	19	(25.7)	0.87
No	32	(58.2)	23	(41.8)	55	(74.3)	
**Healthcare facility opted for injectable treatment**							
Public healthcare	15	(46.9)	17	(53.1)	32	(43.2)	0.67
Private health care	23	(71.9)	9	(28.1)	32	(43.2)	
Not Applicable	5	(50.0)	5	(50.0)	10	(13.6)	

^a^ derived by applying chi-square test with Yate’s correction

^b^ Patients categorized as **Treatment sought** if they reported more than 50% of experienced AEs and **Treatment not sought** if reported less than 50% of experienced AEs

### Qualitative:-

To further explore the factors associated with low treatment seeking or reporting of AEs and to identify challenges from patient’s and health care provider’s perspective, exploratory qualitative interviews were carried out with patients and health care providers.

The perceptions regarding adverse events in DR-TB treatment and treatment seeking behaviour for AE management from patients and health care provider perspective were coded under 12 codes organised into four categories. These four categories were grouped into two broad themes a) Health system related challenges and b) Patient-related challenges and listed in Table 3.

**Table 3:** Perception and challenges regarding reporting of adverse events related to DR-TB treatment from the health providers’ and patient’s perspective

Themes	Sub-theme	Verbatim quotes
**Health system related**		
Provider related	Acceptance and neglect of the AEs	*“Side effects, which will be definitely there, as it is MDR TB, I counselled (my) best to the patients to continue the drug as they will be get used to side effects eventually”(Male, STS, Rural)* *“There is no other treatment regimen available free of cost, patients have to complete course or have to die with TB.” (Male, DOTS provider, Urban)*
	Stigma and discrimination towards patients	*“They are defaulters, they always complain more and adhere less to prescribed medicines” (Male DOTS provider, Urban)* *“When I visited theeye department in district hospital, the nurse asked me to maintain distance from other patients as she realized I am a TB patient.” (Male patient Urban)* *“I always ask them to contact medical facility, but defaulters have tendency to no believe in what we say…” (Male DOTS provider)*
Health care facility related	Poor capacity and no Standard Operating Procedures	*“Whenever, we asked for help regarding side effects, he (DOTS provider) directly refers me to district hospital which is far and not worth attending, they take lots of time and send me from one department to another department.” (Female patient, Urban)* *“She (DOTS provider) sent me to ANM then to PHC and lastly to district hospital, why not (send me) directly there, it took me seven days to get proper care” (Male patient, Rural)* *“I rarely attend any MDR TB cases, they have such a complicated treatment regimen, I always refer them directly to higher centre or give them some antacids, nothing can be done at PHC.”* *(Female MO,PHC Rural)*
	Lack of coordination between DR-TB centre and hospital	*“We know, the patients have to suffer a lot in tertiary care centre for AE management, but we have to send them to concerned department for consultation.” (Male, program manager, Urban)* *“With multiple referrals in government hospitals, patient have to come back without being attended many times due to high load of patients. It would be better to have special OPD for them” (Male, program manager, Urban)*
Program related	Lack of guideline and training for AE identification and management	*“I want to help patients, but I am not sure what I can do at my level for such a disease”(Female DOTS provider-Nurse Urban)* *“We could not decide based on symptoms and history alone whether it is really AE or complication of MDR TB.”(Female PHC-MO Rural)*
	Lack of proper counselling support and empathy	*“Medical Officer has scolded me for my repeated complaint of diarrhea and he has asked me to either continue or stop the medicines. Eventually I learned to stop medicines temporarily whenever I do not feel good” (Male patient, Urban)* *“Doctor had advised me on the first day of treatment that, being defaulter of TB treatment, I have to bear all side effects of higher dose anti-TB drugs”. (Female patient, Rural)*
**Patient related**		
Patient level	Misconceptions	*“Side effects are good in a way which promises early cure.” (Female patient Urban)* *“Side effects are sign of effectiveness of drug which is killing power of Bacteria.”(Male patient Rural)* *“Medicines are free for us, why to complaint for that.” (Female patient Rural)*
	Previous treatment experience	*“I have visited district hospital twice and they took two days for referring me from one department to another for my abdominal pain and lastly, they have given me same tablet which was prescribed by ANM for acidity.” (Female patient Rural)* *“I have visited many times, various clinics, which resulted in no relief. Inspite of that, now I stop taking drugs for few days whenever I do not feel good” (Male patient, Urban)*
	Accessibility and affordability	*“I am already not able to work and I could not further afford visiting (the) hospital (Male patient, Rural)”* *“I asked for help regarding skin rashes and ANM referred me to district hospital which is far and time consuming, I cannot afford to lose my day” (Male patient, Urban)* *“Why to report side effects, doctor will add some more vicious drugs…” (Male patient Urban)*
	Nature of Adverse Event	*I was having tinnitus since so many days but I didn’t bother for that, once I realized my hearing hadreduced, I reported to ASHA the same.* *(Female patient, Rural)*

*DOTS* Directly Observed Treatment Short course, *ASHA* Aaccredited Social Health Activist, *STS* Senior treatment supervisor, *MO-PHC* Medical officer, Primary Health Centre, *ANM* Auxiliary Nursing Midwives

The qualitative findings from the health system and programmatic point of view were explored using manual content analysis to segregate them as per issues related to the providers, health care facility and the program. The health care workers and providers showed an acceptance to presence of AEs during the treatment; which translated to neglecting and/or lack of acknowledgment of their occurrence. Some of the providers also mentioned that since the patients were loss to follow up *(often)*; they find one or the other reason to adhere less to prescribed medication. Stigma, discrimination was thus inherent among the providers. (Table 3).

*“There is no other treatment regimen available free of cost, patients have to complete course or have to die with TB.” (Male, DOTS provider, Urban)*

*“They are defaulters, they always complain more and adhere less to prescribed medicines” (Male DOTS provider, Urban)*

Poor treatment capacity, lack of coordination between the DR TB Centre and the hospital for prompt treatment of AEs and management of the patients; as well as lack of Standard operating procedures (SOPs), guidelines and training for the reporting/ treatment and addressing of AEs were evident health care related and programmatic challenges.

*“With multiple referrals in government hospitals, patient have to come back without being attended many times due to high load of patients. It would be better to have special OPD for them” (Male, program manager, Urban)*

*“We could not decide based on symptoms and history alone whether it is really AE or complication of MDR TB.”(Female PHC-MO Rural)*

The qualitative data suggested lack of awareness among patients regarding importance of management of AE and most common AE. It suggested serious gap in pre-treatment counselling. There were many misconceptions among the patients related to treatment and based on their experience of AE management with previous treatment regimen, they avoided contacting health care provider. This misconception regarding AE management could have been countered with active support from health care providers but as described above; the awareness and attitude towards managing AE was found to be unsatisfactory among health care providers too, which leads AE unattended. It was observed that patients chose to stop medications for a few days to get relief from AEs.

*“Doctor had advised me on the first day of treatment that, being defaulter of TB treatment, I have to bear all side effects of higher dose anti-TB drugs”. (Female patient, Rural)*

*“Side effects are sign of effectiveness of drug which is killing power of Bacteria.”(Male patient Rural)*

*“….….. now I stop taking drugs for few days whenever I do not feel good” (Male patient, Urban)*

Taking medications by missing work due to disease or daily treatment was superimposed with having to take treatment for AE or pay for its management. This led to loss of wages; or fear of additional medications; which may have reduced AE reporting among patients.

*“I asked for help regarding skin rashes and ANM referred me to district hospital which is far and time consuming, I cannot afford to lose my day” (Male patient, Urban)*

These patient related challenges were supported by findings with interview of HCP, as mentioned above, wherein, stigmatizing attitude were quite evident from frontline worker to professionals. Misconceptions regarding DR-TB treatment were also present in HCP. (Table 3).

## Discussion

The present study highlights adverse events (AEs) among patients with DR-TB, one of the most important factors associated with drug adherence in DR-TB [24–26]. All 74 patients experienced at least one AE during the three months of treatment duration; GIT, ophthalmology and otolaryngology organ systems were most commonly involved. The incidence rate of AEs (per 100 person days) during the study period was 3.11 (4.6, 2.7 and 2.02 for the first, second and third month from initiation of treatment respectively).

Findings of our study are relevant and novel for a country like India where rate of MDR and Loss to follow up (LTFU) is very high [2]. Several studies have been conducted on AEs during DR-TB treatment till date which were mainly record-based, but discordance between AEs experienced by patients and reported in clinical records have been reported by various studies for TB as well as other diseases [27–31]. To the best of our knowledge, the present study is the first to assess comprehensively the incidence and pattern of AEs experienced among patients with DR-TB using a patient centered approach, treatment seeking behavior for its management and factors associated with treatment seeking. Mixed methods design of the study is an additional strength of our study which explored the challenges in seeking treatment for AEs and reporting the same from both the patients and treatment providers’ perspective.

The findings related to pattern of AEs are consistent with previous studies [14, 15, 24, 32]. Our study reported higher incidence of AEs compared to other studies conducted in India. This could be due to direct reporting of AEs by the patients rather than extraction of data from records which might have missed these events [9, 10, 33]. However, patients have tendency to over report side effects on being interviewed and this may have contributed to higher incidence.

The higher proportion of AEs occurring in the first month of treatment in this study is in congruence with the pharmacological assumption of increased AEs in the first few months and days of treatment initiation [34]. In studies done elsewhere in India and globally, a higher proportion of AEs were reported in later months of treatment initiation [20, 24, 32, 33]. The likely reason could be a reporting bias as record based studies might have missed the AEs in reported in the initial months either by the patients or by health systems.

In our study, for two fifths of AEs, patients did not contact any health care providers (HCP) for treatment and thus these AEs may have remained unreported in program records. Missed and/or unreported AEs hamper the pharmaco-vigilance system recommended by WHO [35], which focuses on management and prevention of AEs in tuberculosis. Delayed or non-reporting of AEs to health care facility can miss the serious AEs and this can further lead to LTFU or permanent disabilities or death [36]. Regular follow up of patients and specific, targeted enquiry into AEs, as well as careful reporting by HCPs and counsellors, is likely to improve overall treatment outcomes and treatment experience for patients.

None of the demographic, clinical and behavioural characteristics were associated with treatment seeking behaviour. However, qualitative findings reflected various factors like misconceptions about AEs and its management, accessibility and affordability of management, lack of counselling support, past treatment experience being responsible for not seeking care. Furthermore, knowledge regarding adverse events and importance of identification and early management was found to be low; not only in patients, but also in health care providers and these findings are consistent with other studies [37–39]. Misconception regarding tuberculosis and AEs were evident among health care providers; similar to studies conducted in Gujarat and India [38, 40, 41]. It was also evident from interviews with patients that they chose to stop medication for a few days to get relief from AEs which can lead to further development of drug resistance. Affordability and accessibility of higher centre for diagnosis and treatment also plays an essential role in reporting and seeking care as most of the patients belong to lower socio-economic status or lose their job due to TB [42]. The poor socio-economic status of this vulnerable group of patients can also influence the patients’ power to demand the treatment for AE management. Trainings and specific guidelines for identification, management and proper referral for further treatment was the felt need of the HCPs. Qualitative results have suggested stigma and discrimination towards patients with DR-TB among HCPs; which might have lead to stigmatizing approach towards AE management and thus the training of HCPs are need of an hour for providing supportive care to patients with DR-TB.

Our study is not without limitations. The information obtained regarding AE episodes were subjective and not cross-verified with any clinical evidence. However, it has been quoted that self-reported history by patients is the best source to capture any clinical event including AEs [43]. We understand the possibility of recall bias in our study which could be low due to a) interview being done by a doctor; and b) systematic application of questionnaire and c) probing. Interpretation of the study findings needs caution as it studied a small sample size and data came from a single DR TB centre. However, experience of AEs in all patients and low reporting reflects the ground situation which warrants further multi-center studies. In addition, the data is important in the local context in terms of identifying the training needs and counseling approach. Absence of visual clues and probing due to telephonic conversation could have compromised rapport and resulting under/over reporting of AEs is another limitation of our study. However, telephonic conversation provided a better platform to discuss sensitive topics and allowed the health care providers to respond to the programmatic challenges without hesitations. The present study focused on adverse events that were missed by the health system. Our findings could guide policy-makers to sensitize and train all health care providers under RNTCP about the effective solicitation and management of AEs among patients being treated programmatically for MDR TB. Counselling of patients at the time of initiation of treatment regarding AEs and importance of reporting and regular follow up visits and systematic reporting of AE can be included in the treatment approach. Objective and real time assessment of AEs; as well as triangulation of reported and documented AEs can be planned for further practice change by introducing various tool like AE handbook, telephonic counselling etc. The pathway of care for AE can be studied to improve the understanding of treatment seeking behavior of the patients before establishing any independent AE management system in the program. For the effective management of AEs at facility level, start up of special OPD for patients with AEs could be suggested, which can not only be beneficial to provide specialized care and counselling but can also improve patient satisfaction.

## Conclusion

The incidence of AEs was high among patients with DR-TB in the first three months of treatment and treatment seeking/reporting was low. Adequate health education and counselling of the patient and orientation of the health systems specifically for supportive care towards patients with DR-TB are urgently needed. An efficient real-time reporting and management of AEs by developing prospective solicitation (rather than retrospective) can be developed and tested for effective DR-TB treatment and control.

## Supplementary Information

**Additional file 1: Supplementary Table 1.** Operational definitions. **Supplementary Table 2.** Symptoms of adverse events (*n* = 207) experienced by patients during first three months of initiation of DR-TB treatment (during July–September 2018) under the RNTCP at a DR-TB Center, Ahmedabad, Gujarat

**Additional file 2.**

## Data Availability

The datasets used and/or analysed during the current study are available from the corresponding author on reasonable request.

## Abbreviations

AE: Adverse event
ANM: Auxiliary Nursing Midwives
ASHA: Aaccredited Social Health Activist
CBNAAT: Cartridge Based Nucleic Acid Amplification Test
COREQ: Consolidated Criteria for Reporting Qualitative Research
DOTS: Directly Observed Treatment Short course
DR-TB: Drug Resistant Tuberculosis
DST: Drug Sensitivity Testing
HCP: Health Care Provider
ICH: International Council for Harmonisation of Technical Requirements for Pharmaceuticals for Human Use
IRL: Intermediate Reference Laboratory
LPA: Line Probe Assay
LTFU: Loss To Follow Up
MDR: Multi Drug Resistant
OPD: Out Patient Department
PHC: MO-Primary Health Centre Medical Officer
PMDT: Programmatic management of Drug Resistant Tuberculosis
RNTCP: Revised National Tuberculosis Control Program
RR: Rifampicin Resistant
SOP: Standard Operating Procedure
STDC: State Tuberculosis Training and Demonstration Centre
STROBE: strengthening the Reporting of Observational studies in Epidemiology
STS: Senior treatment supervisor
WHO: World Health Organization
