# Supplementary material for: “Side effects--part of the package”: a mixed methods approach to study adverse events among patients being programmatically treated for DR-TB in Gujarat, India
Source: BMC Infect Dis. 2020 Dec 2;20:918. doi: 10.1186/s12879-020-05660-w (PMC7709264; doi:10.1186/s12879-020-05660-w)
Supplement: Supplementary file 1 — Additional file 1: Supplementary Table 1. Operational definitions. Supplementary Table 2. Symptoms of adverse events (n = 207) experienced by patients during first three months of initiation of DR-TB treatment (during July–September 2018) under the RNTCP at a DR-TB Center, Ahmedabad, Gujarat [file 12879_2020_5660_MOESM1_ESM.docx]

| **Table A: Operational definitions (Supplementary table 1)** | |
| --- | --- |
| **DR –TB** | A patient having bacilli that are resistant using phenotypic or genotypic methods to at least Isoniazid or Rifampicin (the DST result being from an RNTCP accredited IRL) |
| **Adverse Event (AE)** | Any untoward medical occurrence in a patient or clinical investigation subject given a pharmaceutical product; does not necessarily have a causal relationship with such treatment.(18) |
| **Loss to follow up (LTFU)** | A patient whose treatment was interrupted for one consecutive month or more.(3) |
| **Transferred out** | A patient who was transferred to another DR-TB center.(3) |
| **Addiction** | Patient reporting to currently consuming any addictive substance (Tobacco chewing/smoking, Alcohol) in any amount or frequency; and consuming the substance for more than a year, and not having quit the same before a year of treatment initiation is termed as having addiction to that particular substance in the context of our study. |
| **Treatment-seeking behavior** | If patient contacted any health care provider for notification or for management of Adverse event experienced after DR-TB treatment, he/she was considered as having sought treatment. The tendency of patients to report/seek treatment for AE is being interpreted as treatment seeking behavior, in the context of our study. |

Supplementary table:-B **(Supplementary table 2)**

Symptoms of adverse events (n=207) experienced by patients during first three months of initiation of DR-TB treatment (during July-September 2018) under the RNTCP at a DR-TB Center, Ahmedabad, Gujarat

| **No** | **System involved** | **Symptom** | **Frequency** | **Percentage** |
| --- | --- | --- | --- | --- |
| 1 | Gastrointestinal | Nausea/vomiting | 21 | 10.1 |
|  |  | Pain in abdomen | 11 | 5.3 |
|  |  | Diarrhea | 9 | 4.3 |
|  |  | Loss of appetite | 15 | 7.2 |
| 2 | Ophthalmology | Pain in eyes | 8 | 3.9 |
|  |  | Diminished vision | 4 | 1.9 |
|  |  | Blurred vision | 3 | 1.4 |
|  |  | Disturbed color vision | 6 | 2.9 |
| 3 | Otolaryngology | Diminished hearing | 6 | 2.9 |
|  |  | Ringing of ear | 7 | 3.4 |
|  |  | Hearing loss | 2 | 1.0 |
|  |  | Balance | 7 | 3.4 |
| 4 | Urinary | Anuria | 1 | 0.5 |
|  |  | Oliguria | 5 | 2.4 |
|  |  | Dark colored urine | 4 | 1.9 |
|  |  | Face swelling | 5 | 2.4 |
|  |  | Leg swelling | 2 | 1.0 |
| 5 | Psychiatric | Suicidal | 4 | 1.9 |
|  |  | Hallucination | 3 | 1.4 |
|  |  | Depression | 2 | 1.0 |
|  |  | Abnormal behavior/thoughts | 3 | 1.4 |
| 6 | Central nervous system | Slowness of activity | 2 | 1.0 |
|  |  | Convulsion | 1 | 0.5 |
| 7 | Hepato-biliary | Yellow skin | 5 | 2.4 |
|  |  | Yellow eye | 5 | 2.4 |
|  |  | Yellow urine | 3 | 1.4 |
| 8 | Peripheral nervous system | Tingling/numbness/burning in lower extremity | 3 | 1.4 |
| 9 | Musculoskeletal system | Pain in joints/tendons/walking difficulty | 6 | 2.9 |
| 10 | Dermatology | Rash | 4 | 1.9 |
|  |  | Itch | 5 | 2.4 |
| 11 | Other | Hair loss | 2 | 1.0 |
|  |  | Oral cavity rash | 1 | 0.5 |
|  |  | Weight gain | 4 | 1.9 |
|  |  | Bitterness of mouth | 2 | 1.0 |
|  |  | Menstruation irregularity | 1 | 0.5 |
|  |  | Lethargy/tiredness | 5 | 2.4 |
|  |  | Dryness of mouth | 7 | 3.4 |
|  |  | Pallor | 7 | 3.4 |
|  |  | Palpitation | 2 | 1.0 |
|  |  | Headache | 7 | 3.4 |
|  |  | Swelling of neck | 2 | 1.0 |
|  |  | Flu | 4 | 1.9 |
|  | Total |  | 207 | 100.0 |
