# Supplementary material for: “Side effects--part of the package”: a mixed methods approach to study adverse events among patients being programmatically treated for DR-TB in Gujarat, India
Source: BMC Infect Dis. 2020 Dec 2;20:918. doi: 10.1186/s12879-020-05660-w (PMC7709264; doi:10.1186/s12879-020-05660-w)
Supplement: Supplementary file 2 — Additional file 2. [file 12879_2020_5660_MOESM2_ESM.docx]

**TB treatment card number:-_____________**

**Date of Interview:- / /20__**

**Date of registration in treatment card:- / /20__**

**Registration date:-**

| **Sr No.** | **Characteristics** | **Options** | **Comments** |
| --- | --- | --- | --- |
| 1 | Age |  |  |
| 2 | Sex | Female  male  Other |  |
| 3 | Residence | Rural  Urban  Slum  Migrant  Prison  other |  |
| 4 | Education | Illiterate  Can read and write  Primary  Secondary  Higher secondary  Graduate  Post graduate and above |  |
| 5 | Occupation | Home-maker  Unemployed  Unskilled  Semi skilled  skilled  Clerical  Semi-professional  professional |  |
| 6 | Total family income monthly |  |  |
| 7 | Total family members |  |  |
| 8 | Co-morbid conditions | HIV  DM  HTN  HD  Renal  Hepatic  Nephro |  |
| 9 | Past history of TB | New case  TB cure  TB rx completed  Tb rx failed  Failure to respond  LTFU  Not evaluated  Rx changed  Not known |  |
| 10 | Addiction | No addiction  Tobacco chewing  Smoking  Alcohol  other |  |
| 11 | Type of drug resistance |  |  |
| 12 | History of intake of drug other than TB | Yes  No  Don’t know |  |
| 13 | Health care facility for daily injection | Sub-centre  Primary Health Centre  Sub District Hospital/District Hospital  Drug resistant TB center  Trust hospital  Unqualified private practitioner clinic  Qualified private practitioner clinic  Other  Not known |  |
| 14 | Health care provider of daily injection | Not applicable  ANM  Private nurses  Unqualified private practitioner  Qualified private Practitioner  Medical officer  TB Medical officer  DOTS provider  Other  Not known |  |
| 15 | Any reported ADR in first month | Yes  No  Not recorded |  |
| 16 | System involved | Not applicable  Gastro Intestinal System  Central Nervous System  Hepatic system  Nephrotic system  Musculo skeletal system  Cutaneous  Endocrine  Psychiatric  Other |  |
| 17 | Symptoms denotes which ADR | Na  Nausea/vomiting  pain in abdomen  burning sensation  diarrhea  Loss of appetite  pain in eyes  diminished vision  blurred vision  disturbed colour vision  Ringing of ear  Diminished hearing  Hearing loss  Balance  Face swelling  Leg swelling  Anuria  Oliguria  Suicidal  Hallucination  Depression  Abnormal behavior/thoughts  Slowness of activity  Convulsion  Yellow skin  yellow eye  Yellow urine  Dark coloured urine  Swelling of neck  Weight gain  Lethargy/tiredness  Tingling/numbness/burning in lower extremity  Dryness of mouth/decreased urine  Pale look  Palpitation  Headache  Pain in joints/tendons/walking difficulty  Rash  Itching  Rash involving oral cavity  Flu |  |
| 18 | Adverse drug event | NA  Ototoxicity  Peripheral Neuritis  Nephroto  Heaptic  Dehydration  Jaundice  All-body  Thyroid  hypersensitivity |  |
| 19 | Episode |  |  |
| 20 | Duration |  |  |
| 21 | Self perceived severity of ADE by patients | Not bothered  Bothered a little  Bothered  Bothered a lot  Not recorded |  |
| 22 | Did you report that ADR | No  Yes  Not recorded |  |
| 23 | First contacted health facility | Same as question number 13  District TB centre  Other  Not known |  |
| 24 | First contacted health care provider | Same as question number 14  TB health visitor  TB Senior TB Supervisor  Dots plus supervisor  District DR TB centre  Other  Not known |  |
| 25 | Management received | counseled  referred  drug changed  advice to stop treatment  supplementary drug  Laboratory investigations  not attended  admitted  other  not known |  |
| The question number 16 to 25 will be repeated for second month history of ADE | | | |
| The question number 16 to 25 will be repeated for third month history of ADE | | | |

**Annexure : 1 In depth Interview of Patients:**

**Interview guide:-**

**Name of the Interviewer:**

**Date of Interview: Interview start / end time:**

**Designation:**

**Centre idea: - To find out patterns of ADE, treatment pathway and enablers and disablers for ADE reporting a treatment seeking (Personal level/social/programmatic level)**

After a brief introduction to the participant regarding the purpose of the interview, written informed consent will be obtained for the interview by PI. Patients will be assured about confidentiality and only PI and participant will be there at time of interview; at a place and time convenient to the patient.

Ice-breaking:-

From when did you start treatment?

Before this have you ever taken treatment for TB?

Since how long you are taking drugs for TB?

What do you know about this disease and treatment?

What do you know about ADE associated with TB drugs?

Have you ever experienced ADE?

From the following, have you experienced any event? [Probe: - checklist with Sign/Symptoms of common ADE]

What was the duration and severity of ADE?

What did you do about it?

[Probe: - when and whom did they contact, where did they go first, duration between ADE experienced and sought treatment, what management did they receive]

If they have not reported ADE, reasons for the same?

[Perception and awareness about ADE, consequences of severe ADE]

What and who made them not report and seek treatment for ADE?

[Personal/Social/provider level factors]

Any experiences related to ADE, reporting and management?

[Probe: - patients will be asked to narrate the description in a timeline manner; as and when the events occurred- time, money involved and pathway to seek care based on experienced ADE]

PI will acknowledge time and input given by patients. Necessary advice and health education will be given at end of the interview. Patient will be advised to refer if needed.

**Annexure: 2 Key informant interviews of health care providers:**

**Interview guide:-**

**Name of the Interviewer:**

**Date of Interview: Interview start / end time:**

**Designation:**

**Centre idea: - To find out enablers and disablers for ADE reporting and treatment seeking (Personal level /programmatic level)**

After a brief introduction to the participant regarding the purpose of the interview, the written informed consent will be obtained for the interview by PI.

1. What are the possible ADEs associated with MDR/RR-TB treatment [Probe: Symptoms, sign]
2. What is your experienced regarding ADE reporting, why do the patients not report? [Probe: programmatic, personal factors]
3. If there is delay in reporting and management, what could be the probable reason? [Probe: severity ]
4. Are there any reasons regarding not reporting ADEs? [Probe: programmatic, patient-level, cost]
5. How can we further improve the identification and management of ADEs?
6. Additional remarks, if any?

**Annexure:-3 FGD of health care providers:**

**Topic guide:-**

**Name of the Interviewer:**

**Date of Interview: Interview start / end time:**

**Designation:**

**Centre idea: - To find out enablers and disablers for ADE reporting and treatment seeking (Patient related and program factors)**

FGD will be conducted at a time and place convenient to the participants. After a brief introduction to the participant regarding the purpose of the interview, the written informed consent will be obtained for the FGD by PI.

Icebreaking:-

How much population do you cater to and how many MDR/RR-TB patients registered?

What do they know about ADE in MDR/RR-TB?

What are the common ADEs have you observed?

Do patients report ADE by themselves?

Where did they go first to report ADEs?

Where do you refer them for ADEs?

Have you had any experiences related ADEs and management?

What do you think what and who made them not to report ADEs?

What can be done to increase reporting of ADEs?

Any suggestions to improve the reporting and/or management of patients?

Participants will be acknowledged for time and input given by them. Summary of FGD will be read back to them.
